# Multislice Spiral CT Imaging Localization and Nursing Care of Catheter Fracture of Scalp Vein Indwelling Needle

**DOI:** 10.1155/2021/9092836

**Published:** 2021-12-17

**Authors:** Wei Liu, Yanhong Zhang, Lifen Zhang

**Affiliations:** ^1^PET/CT-MR Center, Harbin Medical University Cancer Hospital, Haerbin, Heilongjiang 150000, China; ^2^ICU Unit 1, Harbin Medical University Cancer Hospital, Haerbin, Heilongjiang 150000, China; ^3^Pharmacy Intravenous Admixture Service Center, Harbin Medical University Cancer Hospital, Haerbin, Heilongjiang 150000, China

## Abstract

In order to improve the success rate of scalp venous indwelling needles in infants, image positioning and nursing of scalp venous indwelling needle catheters broken with multislice spiral CT were discussed. In this method, three-dimensional reconstruction of multislice spiral CT was used to diagnose and locate the broken catheter according to the anatomical morphology of the indwelling needle, and the treatment and related nursing were carried out. The results showed that the body temperature was 38.7°C, the pulse was 106 times/min, the respiration was 30 times/min, and the body weight was 15 kg. Laboratory examination: the percentage of leukocytes was 10.00 × 10/*L*, the percentage of lymphocytes was 24.8%, and the percentage of neutrophils was 63.7%. Head CT examination: no obvious abnormalities were observed. 31.9% of children diagnosed with hyperfebrile convulsions had good image quality after treatment with MSCT for catheter fracture of scalp vein indwelling needle; good quality was 52.8%, and barely diagnosed was 12.1%. Multiple post-treatment functions of MSCT have important value in the application of three-dimensional localization of foreign bodies in soft tissues in children and play a more important role in the diagnosis and preoperative evaluation of foreign bodies. Appropriate nursing care for children in the image location examination has very important guiding significance.

## 1. Introduction

Scalp needle puncture before intravenous infusion therapy is the top priority of daily pediatric nurses' work. Due to the particularity of infants' physiology, small blood vessels, and difficult puncture, children are afraid, active, and uncooperative. In addition, some parents often impose a variety of bad words and deeds during the puncture, which causes great psychological pressure on the nurses who are performing infusion operations. Inexperienced young parents often pull out the needle or touch the needle to cause fluid extravasation because they are not good at caring for the children who are undergoing infusion. This not only intensifies the bad emotions of parents but also increases the labor intensity of our pediatric nurses [1]. In order to reduce the damage to the peripheral vascular system of children, reduce the pain caused by repeated puncture, unnecessary links in the process of infusion and the resulting disputes, reflecting the people-oriented service purpose, the intravenous indwelling needle, as advanced new infusion equipment, has been widely used in clinical infusion because of its simple operation, soft cannulation, and long indwelling time in the vein [2]. However, in clinical practice, there is not an assistant for every operation, and more often, it is performed by one operator after the successful recovery of blood by indwelling needle puncture. The fixation of infants' heads is the key to the successful insertion of the indwelling needle hose. The failure of an indwelling intravenous catheter, on the contrary, increases the pain of children [3].With the continuous development of multislice spiral CT (MSCT) and the continuous maturity of technology, coronary CT angiography (CTA) has become the main means of pretreatment screening for coronary atherosclerotic heart disease (coronary heart disease). This convenient and noninvasive examination method has been recognized by most physicians and patients. The success rate and image quality of the coronary artery CTA examination are affected by many factors, such as heart rate, choice of gating mode, total amount of contrast agent, injection flow rate, delay time, and exposure parameters. In daily examination, we found that different types of intravenous indwelling needles also have an impact on image quality. In view of this research problem, Li, L. et al. proposed that scalp vein indwelling needles should be more widely used in clinical practice, and sealing fluid is the key to maintaining patencies. In order to reduce blockage, it is best to choose 25 heparin/ml of normal saline as the sealing fluid, but for diseases such as thrombocytopenia, hemophilia, and children allergic to hepatologists, heparin should not be used, normal saline can be used as a sealing fluid [4]. According to Keiichi et al., the incidence of tube plugging was significantly reduced (17%) by pushing the sealing liquid while retreating the needle, compared with pulling the needle out after the injection (inserting the needle into the trocar 3 mm ∼ SMLN). Because if all the sealing needles are inserted into the sleeve and the sealing fluid is withdrawn from the needle after injection, the blood will flow back into the sleeve with the negative pressure of the drawing needle, leading to blood coagulation and tube blocking [5]. Singh et al. believed that when using the indwelling needle after sealing the tube, attention should be paid to first withdraw blood, and only after blood is recovered can the scalp needle be connected for fluid replacement. It is not appropriate to push the blood clot into the blood vessel forcefully with a syringe to avoid tube blockage [6]. On the basis of the current research, this paper discusses the image positioning and nursing care of the broken catheter of the scalp vein indwelling needle with multislice spiral CT. The method specifically adopts the three-dimensional reconstruction of multislice spiral CT. According to the X-ray anatomy of the indwelling needle, the broken catheter is diagnosed, located, and treated, and related to nursing care. Multiple postprocessing functions of MSCT are of great value in the application of three-dimensional localization of soft tissue foreign bodies in children and play a more important role in the diagnosis and preoperative evaluation of foreign bodies.

## 2. Methods

### 2.1. Object

Male, 1 year and 4 months old. Convulsions occurred while playing, characterized by eyes rolled up, foaming at the mouth, stiff limbs, and loss of consciousness. A 38.7°C body temperature, a pulse rate of 106 times/min, a breathing rate of 30 times/min, and a weight of 15 kg. Laboratory examination: the percentage of leukocytes was 10.00 × 10/*L*, the percentage of lymphocytes was 24.8%, and the percentage of neutrophils was 63.7%. A CT scan of the skull showed no obvious abnormalities. Check the results shown in Table 1. He was diagnosed with febrile convulsion and treated with anti-infection and nourishing brain cells. After 3 days of treatment, the clinical symptoms of the child improved, and the family required the child to be discharged from hospital. After the removal of the indwelling needle, it was found that the catheter of the indwelling needle was broken in the scalp vein, and then the indwelling needle was inserted into the right dorsal foot vein to establish a vein channel [7].

### 2.2. Check Methods

Using Siemens Somatom Definition 64-layer spiral CT, application of full automatic intelligent current selection techniques (ACSs) from the lower jaw to the conventional location and parietal scanning [8]. After the scan is completed, the automatic reconstruction data will be transmitted to the SyngommvvpVE23 A workstation, and the image will be reconstructed jointly with InSpace software and NenroDSA software. It includes multilayer reconstruction (MPR), volume reconstruction (vR), and stereosynthesis with cutting, rotating, and threshold tissue removal techniques. An independent analysis was performed by two experienced associate chief physicians to determine the presence and size of an indwelling needle catheter in the broken vessel.

### 2.3. Removing the Broken Needle

Combined with MPR cross section and sagittal plane recombination image positioning, after basic anesthesia and routine disinfection, a 5 mm small incision was made from the catheter between the orbital medial canthus and the nasal bone closest to the skin. The inner canthus vein was separated and exposed layer by layer. The broken indwelling needle catheter was gently removed, the wound was closed layer by layer after adequate irrigation and hemostatic, and then the patient was returned to the ward [9].

## 3. Results and Analysis

### 3.1. Cause Analysis of Fracture of Intravenous Indwelling Needle in Children

Because the operation of the intravenous indwelling needle is simple, the cannula is soft, and it is not easy to penetrate the wall of the vessel when it is placed in the vessel; the vessel is protected, and the pain caused by repeated puncture of the vessel is reduced, thereby reducing the nursing workload. In particular, the application of superficial venous indwelling needles for infusion in children is convenient, safe, quick, and effective, so it is favored by clinical nursing staff [10]. Scalp veins are generally selected for indwelling needles in children. The longest time of scalp indwelling is followed by the dorsal hand and dorsal foot veins. There is no uniform standard for the indwelling time of the indwelling needle. The nursing standard of the Intravenous Infusion Association stipulates that the indwelling time is 72 h, and the instructions for the use of domestic indwelling needles suggest that the indwelling time should not exceed 72–96 h. In this study, a case of pediatric scalp venous indwelling needle was reported for 72 h, and the catheter fracture of the indwelling needle was not caused by the indwelling time [11]. The possible reasons are as follows: (1) children themselves are active factors, leading to the scalp indwelling needle catheter and catheter seat shaking; (2) children with fever sweat easily after fever reduction, resulting in the sterile transparent paste not being firmly fixed; (3) sealing or infusion connection, resulting in the loosening of the indwelling needle; and (4) touch in the process of feeding children, resulting in the shaking of the indwelling needle catheter and catheter seat. Therefore, the use of a scalp vein indwelling needle in children requires nurses to have skilled skills, a high sense of responsibility, and good communication skills; at the same time, parents should also be guided not to touch the needle seat and sealed infusion connector to avoid breaking the indwelling needle catheter.

### 3.2. Advantages of Multislice Spiral CT (MSCT) in Locating Nonmetallic Foreign Bodies

The imaging methods for locating foreign bodies in soft tissues include ordinary *X*-ray, ultrasound, CT, and MRI. Ordinary X-ray film can show high-density foreign bodies; most foreign bodies can be displayed in an ultrasound examination. Because of its low resolution, it is difficult to display nonmetallic foreign bodies in children's soft tissues, and it is rarely used in clinics. MRI examination can implement multiazimuth scanning imaging and three-dimensional reconstruction, but the scanning time is long, and children are not easy to cooperate with. For small foreign bodies close to soft tissue, it is easy to miss the diagnosis. Conventional CT cannot carry out multiplane restructuring and 3D reconstruction, nor can it carry out multiazimuth observation, so it is difficult to display small foreign bodies in children's soft tissues [12]. The 64-slice spiral CT scan has thin layers and can be reconstructed in multiple planes. MPR can be cut in any plane, and VR's false color 3D reconstruction image is intuitive and three-dimensional and can be rotated in any direction, which is welcomed by clinicians. Therefore, it is widely used in locating foreign bodies in soft tissues in children. The fractured catheter of an intravenous indwelling needle in children was scanned and recombined with 64-slice helical CT. Not only the position of the catheter but also the distance between the catheter and the skin surface could be clearly shown in cross section, sagittal plane, and coronal plane of MPR. VR reconstructed images can stereoscopically reproduce the shape of the catheter broken by the indwelling needle, providing a good positioning basis for surgical removal [13].

### 3.3. MSCT Technology and Image Postprocessing

**Figure 1:**
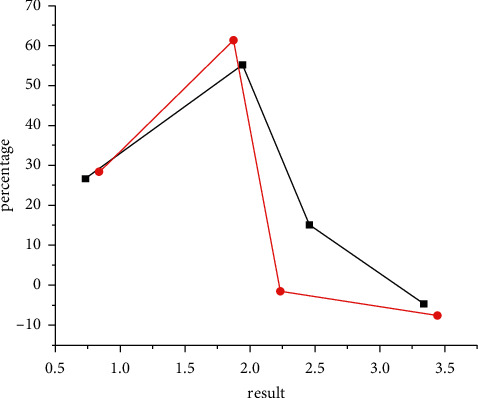
Comparison of the image quality of children at different periods.

The high-speed 64-slice spiral CT scan can effectively overcome the motion artifacts and can also complete the examination under the conditions of spontaneous breathing and centerless gating, which is very suitable for pediatric patients. The vital organs and functions of children have not yet been fully developed, and all organs are sensitive to radiation. Excessive X-ray radiation does great harm to children and increases the risk of cancer. Therefore, how to develop appropriate scanning parameters is a hot research topic at present. The same mAs value is not appropriate due to the differences in size and absorption of X-rays between children. The ACS technology is used in the multislice spiral CT examination in children, and the ASC technology can automatically recommend the best mAs value according to the positioning phase, which not only does not affect the image quality but also avoids dose waste occurrences so that the CT examination scheme is optimized. 64-slice spiral CT has made a greater breakthrough in technology, with a powerful postprocessing function and a good 3D sense of multiplane three-dimensional recombination image, it can construct the whole anatomical picture of the position of the intravenous indwelling needle and broken catheter, and provide clinicians with more intuitive graphics so as to carry out multiangle and multidirectional stereoscopic observation. It provides more information for operation and has the advantages of accurate positioning, safe extraction, time saving, and pain reduction.  Figure 1 is the comparison of the image quality of children in different periods.

### 3.4. Imaging Related Nursing

#### 3.4.1. Preparation before Inspection

① Sedation and braking: In order to prevent the child's body from twisting back and forth during the scan, which will affect the examination, the child should be sedated and braked. For persons under 3 years of age, the sedative 10% chloral hydrate is administered either orally or by retention enema. This medicine is absorbed quickly, the side effects are small, and the maintenance time is long (about 3∼4h), general as the first choice. For patients with poor oral efficacy, intramuscular injection of ketamine (5mgkg) can be used. After injection, lie prostrate and closely observe the vital signs such as pulse, respiration, and blood pressure. ② Psychological nursing: For children who can cooperate with hypnotic drugs as far as possible, psychological nursing should be carried out before the examination [14]. Patience to do a good job of interpretation, to explain to the children the importance of paying attention to the examination and the need to cooperate. Children who have been in hospital for a long time because of long-term injections, medicine, pain, and psychological fear of medical staff, should be told that the examination does not require injections, pain, and discomfort. Scan when the sound, like a train or a car, to eliminate the emotions of children. For the timid children, let them listen to the amplified sound when the machine scans, become familiar with the environment around the machine, and visit other children after the CT examination to relieve the fear. In addition, before the examination begins, the ears of children can be plugged with cotton to reduce the adverse stimulation of scanning. At the same time, during the scanning process, the nurse or the parents of the children accompanied the bedside, holding the children with their hands so that the children have a sense of security. If possible, ask the parents to put the child to sleep.

#### 3.4.2. Coordination during Inspection

While X-rays provide convenience for clinical diagnosis and treatment, they also cause damage to the human body, especially to children, such as mental decline and growth retardation in infants and young children. Because of the risk of leukemia and cancer, it is more important to protect children from X-ray examination. Children with active cell division, a long life cycle, and sensitivity to X-rays are more likely to develop radiation-induced cancer, and the younger they are, the higher the risk [15]. Pay great attention to shielding the nondiagnostic parts, and focus on protecting the parts sensitive to radiation. Pay attention to covering the neck, chest, and upper abdomen with lead skin, cover the testicles of male children with special lead skin, and the ovary of female children with lead scarf. Use lead scarf to cover the neck and eyes of children, to prevent eye lens, thyroid, gonad, and other sensitive parts from unnecessary exposure.

#### 3.4.3. Health Education after Examination

① Keep away from radioactive sources: ask parents not to lead children to wait in the examination room, and leave the examination room immediately after the examination, in order to reduce unnecessary radiation. Put ionizing radiation signs on the equipment room to make people aware of the danger and keep children away. The machine performance and functions of the department are introduced in eye-catching places so that clinicians and their families know the significance of the examination. ② Diet: children after X-ray examination should eat more food that can enhance the body's resistance, such as mushrooms, agaric fungus, and kelp, take more vitamins, and eat more protein-rich foods to prevent radiation damage, such as eggs, milk, bone soup, fish, fresh fruits, vegetables, and so on. ③ Strengthen the care of catheterization needle: children are born active, and during illness, the head of external objects will produce a sense of curiosity or boredom. Therefore, children's parents and family members should pay more attention to the children's attention to other things. The nursing staff should fix it properly in a way that it does not affect the child's activity as well as the indwelling needle in the functional position. In addition to the use of indwelling needles, special films, and tape with the same fixation, according to the situation, a hat, entrain card, and so on can be worn. For easy sweating and washing, attention should be paid to the shedding and deformation of the indwelling needle [15]. In addition, if the patient's scalp indwelling needle seals the tube again after infusion, the nurse should first check the position of the indwelling needle, check the connection between the needle tube (needle wing) and the base, timely find the problem, timely correct or replace it.

### 3.5. Advantages of Using Indwelling Needles

This group of cases was compared with the previous 154 patients in our hospital as reference data.

#### 3.5.1. Improve the Success Rate of Puncture

MSCT high-pressure enhanced injection is characterized by high pressure, fast flow rate, and injection of a large dose of contrast agent in a short time to form a mass injection, and the contrast agent is thick and not easy to be injected. Large blood vessels and bulky needles must be selected during injection, which brings certain difficulties to the puncture technique [16]. The steel core of the intravenous indwelling needle is small in diameter and the bevel of the needle is small and short, which can reduce the risk of punctured blood vessel wall during puncture. In addition, there is a side hole opening near the indwelling needle tip, which can quickly see blood recovery in the shortest time so as to judge the success of the puncture as soon as possible. In addition, the plastic sleeve outside the needle core is soft, the tube wall is thin, and the tube cavity is large, which can reduce the injection resistance and ensure that there is a large flow of contrast agent in the unit time. Its good performance and safety improve the success rate of punctures [17].  Table 2 compares the success rate of intravenous catheter indwelling between the two groups.

#### 3.5.2. Improve the Success Rate of Enhanced Injection

(1) The examination of some parts (such as the chest, abdomen, and spine) requires the lifting of the upper limb and flexion of the forearm, so as to avoid the influence of bone artifacts on the image quality. The flexion of the forearm can enable the body to enter and exit the scanning hole smoothly during the scanning [18]. Enhancement usually requires an intravenous injection in the upper extremum, while the scanning bed moves in and out of the scan hole during the scan, and because of harmful radiation, delay time, and other factors such as short-term injection and scan almost simultaneously, the staff cannot observe in the field and high-pressure injection cannot be found to contrast leakage in a timely manner. (2)The indwelling needle trocar has good flexibility in the blood vessel, strong vascular compliance, close connection with the blood vessel wall at the puncture point, and can change with the body position without destroying the blood vessel. It avoids the risk of contrast agent leakage into the tissue caused by nontechnical operations during high-pressure injection that cannot reach the scanning site, which reduces the pain of patients and improves the success rate of injection. (3) Maintain a comfortable posture to improve the quality of scanning images. The tube of the indwelling needle can change with position, allowing the patient to remain comfortably positioned until the examination is completed successfully, especially for patients with poor tolerance. Since the examination requires a long time to maintain a fixed posture to avoid motion artifacts, a comfortable posture can prevent the patient from moving and thus improve the image quality [19]. (4) It is an allergic reaction drug conducive to rescue. Because of the toxic and side effects of the contrast agent, some patients still have different degrees of allergic reactions, although the skin test is negative. The indwelling needle is equal to preserving an open vein access, which can facilitate the rescue of medication and provide a time guarantee for saving the lives of patients. (5) The indwelling needle floats in the blood vessel due to the softening function of the cannula material, and the patient feels comfortable during the cannula indwelling. The psychological fear caused by repeated punctures is eliminated, and the indwelling needle puncture has little damage to the tissue. The needle eye is small and easy to stop bleeding, and the pain is slight. In addition, the closed venous indwelling needle can be connected with the extension tube or infusion set before puncture. When the needle core is withdrawn, the white isolation plug automatically closes, which can avoid blood pollution.  Table 3 shows the comparison between the sealing time and the indwelling time [20].

## 4. Conclusions

This paper discusses the imaging localization and nursing care of the catheter fracture of the scalp vein indwelling needle with multi-slice spiral CT. According to the X-ray anatomy of the indwelling needle, the broken catheter was diagnosed, located, treated, and nursed. The results showed that the body temperature was 38.7°C, the pulse was 106 times/min, the respiration was 30 times/min, and the body weight was 15 kg. Laboratory examination showed that WBC was 10.00 × 10/*L*, lymphocyte percentage was 24.8%, and neutrophil percentage was 63.7%. CT scan of the skull showed no obvious abnormalities. In children diagnosed with hyperfebrile convulsion, 31.9% of the images were of good quality, 52.8% were of good quality, and 12.1% of the children barely diagnosed were treated with MSCT for catheter fracture of the scalp venous indwelling needle. The multiple postprocessing functions of MSCT are of great value in the application of three-dimensional localization of soft tissue foreign bodies in children and play a more important role in the diagnosis and preoperative evaluation of foreign bodies. Appropriate nursing care for children in the image location examination has very important guiding significance. In the future, there will be many factors that affect the success or failure of coronary artery CTA examinations and the imaging quality, and the analysis of various influencing factors is helpful for the better development and application of this technology.

## Figures and Tables

**Table 1:** Data table of check items.

Check the project	Body temperature (°C)	Pulse rate	Breathing	Weight (kg)	White blood cells	Lymphocytes	Neutrophils
Data	38.7	106 times/min	30 times/min	15	10.00 × 10/L	24.8%	63.7％

**Table 2:** Comparison of the success rate of intravenous catheter indwelling between the two groups.

Group	Number of cases	Successful	Failure
Control group	74	50 (67.57%)	24 (32.43%)
Observation group	80	70 (87.5%)	10 (12.5%)

**Table 3:** Comparison between the sealing time and indwelling time of group 2.

Group	Number of cases	Tube sealing time	Indwelling time
Control group	74	15.42	95.83
Observation group	80	15.57	95.88
T		1.279	0.139

## Data Availability

The data used to support the findings of this study are available from the corresponding author upon request.
